# Survival and Mortality Predictors in Prostate Cancer: A Retrospective Cohort of 10 556 Brazilian Patients

**DOI:** 10.1002/cnr2.70667

**Published:** 2026-08-31

**Authors:** Wesley Rocha Grippa, Larissa Soares Dell'Antonio, Cristiano Soares Dell'Antônio, Luciane Bresciani Salaroli, Luís Carlos Lopes‐Júnior

**Affiliations:** ^1^ Graduate Program in Public Health, Health Sciences Center Federal University of Espírito Santo (UFES) Vitoria Espírito Santo Brazil; ^2^ Department of Applied Mathematics Northern Espirito Santo University Center at the Federal University of Espírito Santo (UFES) São Mateus ES Brazil; ^3^ Secretaria de Estado da Saúde Do Espírito Santo, Special Epidemiological Surveillance Nucleus, Instituto Capixaba de Ensino Pesquisa e Inovação (ICEPi) Vitória Espírito Santo Brazil; ^4^ Department of Noncommunicable Diseases (NCDs) and Mental Health (NMH) Pan American Health Organization–PAHO Washington DC USA

## Abstract

**Background:**

Prostate cancer (PCa) remains one of the most frequently diagnosed malignancies worldwide and represents a major contributor to premature mortality among men. This study aimed to estimate survival and investigate factors associated with PCa‐specific mortality among men treated within the oncology care network of a Brazilian state.

**Material and Methods:**

Retrospective cohort study was conducted using data obtained from hospital‐based cancer registries linked to the state Mortality Information System. The cohort included 10 556 men diagnosed with PCa between 2000 and 2016, with follow‐up through 2021. Outcomes were categorized as death from PCa, death from other causes, or alive at the end of follow‐up. Associations with PCa‐specific mortality were examined using cause‐specific Cox proportional hazards regression models.

**Results:**

At the end of 2021, 6388 patients were alive, 1936 had died from PCa and 2232 deaths from other causes. The 5‐year PCa‐specific survival was 87.7%. In the multivariable analysis, increasing age at diagnosis (per 10‐year increment) was associated with a higher cause‐specific hazard of PCa mortality (HR = 1.14; 95% CI: 1.06–1.22). Lower educational level was also associated with increased mortality, while patients with basic education (HR = 0.82; 95% CI: 0.71–0.95) and middle or high education (HR = 0.64; 95% CI: 0.52–0.80) showed a lower risk of death compared to those with no formal education. Referral from non‐SUS services was associated with a slightly higher risk of PCa mortality (HR = 1.14; 95% CI: 1.00–1.30). Regarding treatment variables, surgery was associated with a lower cause‐specific hazard of mortality (HR = 0.41; 95% CI: 0.33–0.52), whereas hormone therapy was associated with an increased cause‐specific hazard of death (HR = 1.98; 95% CI: 1.75–2.24).

**Conclusions:**

Age, educational level, presence of distant metastasis, referral source, and treatment modalities were associated with PCa‐specific mortality. However, treatment‐related associations should be interpreted with caution, as they likely reflect underlying disease severity and treatment selection patterns rather than causal effects. These findings highlight the importance of early diagnosis, reducing social inequalities in access to care, and strengthening oncology care networks to improve outcomes in patients with PCa.

## 1 Introduction

Prostate cancer (PCa) is one of the most commonly diagnosed malignancies worldwide and remains a major cause of cancer‐related mortality in men [1]. Globally, approximately 1.4 million new cases and more than 375 000 deaths were estimated in 2020, making PCa the second most frequently diagnosed cancer and the fifth leading cause of cancer death among men [2].

In Brazil, according to estimates from the National Cancer Institute (INCA), PCa is currently the most frequently diagnosed malignancy among men when non‐melanoma skin cancer is excluded, with nearly 78 000 new cases expected annually in the coming years [3].

Over recent decades, substantial advances in cancer diagnosis and treatment have contributed to improved survival rates for several malignancies [4]. In PCa, favorable outcomes are strongly associated with timely diagnosis, rapid access to treatment, advances in therapeutic technologies, and personalized clinical management [4, 5].

Currently, the 5‐year relative survival rate for neoplasms depends on staging, early diagnosis, and the care and support provided by reference centers or units for timely and effective cancer treatment [5, 6]. Despite advances in diagnosis and treatment in recent years, factors such as population aging and the adoption of lifestyles associated with the development of cancer in countries of all income levels mean that both the incidence and mortality from malignant neoplasms continue to increase [4, 7, 8, 9].

For PCa, several risk factors are well established, including advanced age, ethnicity, genetic predisposition, family history of cancer, and hormonal factors [7, 8, 10]. In addition, environmental exposures have gained increasing attention, particularly, pesticide exposure, which has been associated with both PCa incidence and mortality in recent large‐scale studies [11, 12, 13].

There is evidence that PCa indicators vary by place of residence in different populations and geographies [14, 15]. Previous studies on cohorts of patients diagnosed with PCa have sought to understand how risk factors affect survival rates during a given period [16, 17, 18, 19], however, in low‐ and middle‐income countries, such as Brazil, there are few studies that show such associations of risk factors with PCa survival [20, 21], mainly based on data from hospital cancer registries (HCRs).

As part of an ongoing population‐based research program investigating long‐term PCa outcomes in southeastern Brazil, we recently analyzed this retrospective cohort using a competing‐risk framework based on Fine–Gray regression to estimate the cumulative incidence of PCa‐specific mortality while accounting for deaths from other causes [22]. That study focused on estimating the absolute probability of PCa‐specific death in the presence of competing events, providing clinically relevant measures of cumulative risk [23].

The present study was designed to address a complementary but distinct scientific question. Rather than estimating cumulative incidence, we investigated prognostic factors associated with the cause‐specific hazard of PCa mortality using cause‐specific survival analysis. Unlike competing‐risk regression, cause‐specific Cox proportional hazards models estimate the instantaneous hazard of PCa death among individuals who remain at risk throughout follow‐up, making them, particularly, appropriate for etiological inference and prognostic evaluation. Therefore, although both studies were conducted using the same population‐based cohort, they provide complementary rather than overlapping evidence regarding PCa prognosis.

In this context, this study aimed to estimate survival and investigate factors associated with PCa‐specific mortality among men treated within the oncology care network of a Brazilian state.

## 2 Materials and Methods

### 2.1 Study Design and Participants

A retrospective observational cohort study was conducted using a secondary database of the HCR of the State of Espírito Santo (ES) and linkage with the Mortality Information System (SIM) was used. Secondary data were obtained from the Cancer Surveillance of the State Health Department of Espírito Santo (SESA/ES).

The Cancer Care Network of ES is organized into three health regions (North/Central, Metropolitan, and South) [23, 24], and includes one High Complexity Oncology Center (CACON) and seven High Complexity Oncology Care Units (UNACONs) accredited by the Brazilian Ministry of Health. All oncology referral hospitals within the state maintain structured HCRs and annually submit their data to the national Hospital Cancer Registry Integration System (SIS‐RHC) [25, 26].

### 2.2 Data Collection

Initially, a total of 10 560 records corresponding to patients diagnosed with PCa between 2000 and 2016 were retrieved from the HCR database. Four records were excluded because age at diagnosis was coded as 0 or 999, indicating invalid or missing information. The final cohort comprised 10 556 men aged 18 years or older diagnosed with PCa (ICD‐10 code C61) who received care at hospitals belonging to the oncology care network of Espírito Santo and were registered in the SIS‐RHC between January 1, 2000, and December 31, 2016.

Patients were followed until death from any cause or administrative censoring on December 31, 2021, whichever occurred first. Therefore, the study population consisted of 10 556 men diagnosed with PCa and treated within the state oncology network during the study period.

Data extraction was performed between June and October 2023. All datasets were fully de‐identified before analysis, and the investigators had no access to personally identifiable participant information at any stage of the study.

### 2.3 Data Linkage Procedures

Data linkage between the HCR and the SIM datasets was performed using a set of key identifying variables, including patient name, mother's name, date of birth, sex, and municipality of residence [27].

Before linkage, both databases underwent preprocessing procedures aimed at improving record consistency and matching accuracy. These procedures included removal of duplicate records, standardization of variable formats, normalization of dates, and application of phonetic encoding algorithms (Soundex) to name fields [22, 27, 28].

Records linkage was subsequently carried out according to predefined deterministic matching rules. Only records demonstrating exact agreement across the selected identifiers were retained as valid pairs. To ensure linkage reliability, all matched pairs were additionally reviewed manually using complementary information, including residential address, date of diagnosis, and date of death [27, 28].

The HCR is an institutional system designed for the continuous collection, storage, processing, analysis, and dissemination of data related to patients diagnosed and treated for malignant neoplasms within hospital settings [29]. Data collection is based on a standardized Tumor Registration Form developed by the INCA and recognized by the International Agency for Research on Cancer (IARC/WHO).

This form, called the Tumor Registration Form, contains 44 items related to the sociodemographic, tumor, clinical, treatment, and outcome characterization of the case, of which 35 items are mandatory and 9 items are optional [29].

The HCR Tumor Registration Form is used to systematically extract information from medical records, generate standardized case summaries, and support data entry into the SIS‐RHC electronic database system [28]. The structure and content of this instrument are defined according to the informational requirements of hospitals that maintain HCRs [29].

Baseline data were collected, including the following variables obtained directly from the tumor registration form of the HCR of the ES Cancer Care Network, namely Date of diagnosis, age, race/color (White and non‐White), marital status (married and nonmarried), level of education (no education, basic education, middle or high education), state of residence, referral source (Unified Health System or *Sistema Único de Saúde*—SUS and non‐SUS), diagnosis and previous treatment (refers to the patient's oncologic history for the prostate tumor itself and indicates whether the individual had already been diagnosed and/or treated for PCa before being registered at the current hospital), most common basis important for the diagnosis of the tumor, histological type of primary tumor, presence of more than one primary tumor (refers to the presence of another distinct primary malignant neoplasm recorded in the HCR), clinical staging by group (TNM), presence of distant metastases, first treatment received in the hospital (surgery, radiotherapy, hormone therapy, radiotherapy combined with hormone therapy), disease status at the end of the first treatment, type and date of death. The index date used to initiate follow‐up was the date of diagnosis recorded in the HCR.

### 2.4 Statistical Analysis

Sample characteristics were presented as absolute and relative frequencies. Overall and stratified 5‐year PCa‐specific survival, along with their respective 95% confidence intervals (95% CI) were estimated using the Kaplan–Meier method and compared using the log‐rank test [30].

Because deaths from causes other than PCa may act as competing events, Kaplan–Meier estimates should be interpreted as descriptive cause‐specific survival estimates rather than direct cumulative incidence estimates. In this study, Kaplan–Meier curves were used for descriptive visualization of PCa‐specific survival across exposure groups. Etiological associations with PCa‐specific mortality were assessed using cause‐specific Cox proportional hazards models [31]. This analytical approach was selected because the primary objective of the study was to investigate prognostic factors associated with the instantaneous hazard of PCa‐specific mortality rather than to estimate cumulative incidence in the presence of competing events.

This analytical strategy differs from the competing‐risk framework previously reported in this population‐based cohort. Whereas the previous study used Fine–Gray regression to estimate the cumulative incidence of PCa‐specific death while accounting for competing causes of death [22], the present analysis employed cause‐specific Cox regression to estimate the instantaneous hazard of PCa‐specific mortality among individuals who remained at risk throughout follow‐up. Consequently, these two analytical approaches address distinct epidemiological questions and provide complementary rather than overlapping information regarding prognostic factors associated with PCa mortality.

Age at diagnosis was modeled as a continuous variable (per 10‐year increment) in the multivariable analysis to preserve statistical power and avoid arbitrary categorization. For Kaplan–Meier analyses, age was categorized (≤ 65 vs. > 65 years) to facilitate visual interpretation and comparison between clinically relevant groups.

The proportional hazards assumption was formally assessed for all covariates included in the multivariable Cox model using Schoenfeld residual tests, including both global and variable‐specific tests [32]. Graphical inspection of survival curves was used as a complementary diagnostic approach.

Referral source (public health system—SUS vs. private/insurance) was evaluated for inclusion in the multivariable model. Based on Schoenfeld residual testing, no violation of the proportional hazards assumption was identified, and the variable was retained in the final model. A sensitivity analysis stratified by referral source was also conducted to assess the robustness of the findings.

Missing data were assessed descriptively across all variables. A substantial proportion of missingness was observed in key clinical variables, particularly TNM staging and disease status after treatment. The pattern of missingness suggested dependence on clinical documentation processes rather than a completely random mechanism. Given the high proportion of missing data and the absence of strong auxiliary variables to support reliable imputation, multiple imputation was not performed. Instead, a complete‐case analysis was used for the multivariable Cox regression.

To evaluate potential selection bias associated with the complete‐case analysis, a sensitivity analysis was performed comparing baseline demographic, clinical, treatment, and outcome characteristics between patients included in the multivariable Cox regression models (complete‐case sample) and those excluded due to missing data. Continuous variables were compared using Student's *t*‐test or Mann–Whitney *U* test, according to data distribution, whereas categorical variables were compared using Pearson's *χ* ^2^ test or Fisher's exact test when appropriate. In addition to *p*‐values, standardized mean differences (SMDs) were calculated to quantify the magnitude of imbalance between groups independently of sample size. Based on previously established methodological recommendations, SMD values < 0.10 were considered indicative of negligible imbalance, values between 0.10 and 0.20 of small imbalance, and values > 0.20 of meaningful imbalance [33, 34].

All statistical analyses were conducted using R software (version 4.3.1). A *p*‐value < 0.05 was considered statistically significant.

### 2.5 Ethical Considerations

This study was approved by the Research Ethics Committee of the Universidade Federal do Espírito Santo (Ufes) (approval ID: 5.533.541). Authorization to access and use secondary restricted‐access data was also granted by the SESA/ES, Brazil. The requirement for informed consent was waived by the ethics committee because the study was based exclusively on retrospective secondary data from a long historical series. Many of the individuals included in the dataset had either relocated or passed away, making it impossible to contact them. In addition, authorization for the collection of secondary and restricted data for this research was also obtained from SESA/ES.

## 3 Results

The study cohort comprises 10 556 patients with PCa registered in the HCR of the ES state, diagnosed between 2000 and 2016.

Table 1 presents the sociodemographic and clinical characteristics of the sample selected for this study, where the mean age was 69.20 years, with a standard deviation of 9.10 years; the youngest patient was 18 years old and the oldest was 103 years old. 70.39% of the patients with this neoplasm are at least 65 years old, 63.41% declare themselves as non‐white (black, brown, yellow, and indigenous), 65.92% are married, 63.64% are illiterate (no education) or have only basic education, and 97.38% are residents of the State of Espírito Santo. Among the clinical variables, 66.76% are referred by SUS, with a primary tumor in the prostate and without the occurrence of other primary tumors (95.20%), with diagnosis and without previous treatments (62.10%). The histology of the primary tumor was the most important basis for the diagnosis of the tumor in 97.77%, with adenocarcinoma being the most identified histological type of primary tumor in 98.52%. In 91.65% of cases, there were no distant metastases, and the first treatment received in the hospital was surgery in 23.71%, hormone therapy in 21.97%, radiotherapy in 19.47%, and radiotherapy combined with hormone therapy in 15.15% of cases.

**TABLE 1:** Baseline characteristics of all included prostate cancer (PCa) patients (*n* = 10 556).

Variable	*N*	%
Age at diagnosis (in years)		
Mean (standard deviation)	69.2 (9.10)	—
Median (interquartile range)	70 (63–76)	—
Age range at diagnosis		
18–64 years	3126	29.61
65 years or older	7430	70.39
Race/skin color		
White	3010	28.51
No White	6694	63.41
Missing data	852	8.07
Marital status		
Married	6959	65.92
No married	3065	29.04
Missing data	532	5.04
Education level		
No education	1213	11.49
Basic education	5505	52.15
Middle or high education	1238	11.73
Missing data	2600	24.63
State of residence		
Espírito Santo	10 279	97.38
Other state	277	2.62
Referral source		
SUS	7047	66.76
No SUS	1835	17.38
Missing data	1674	15.86
Previous diagnosis and treatment		
No diagnosis/no treatment	2174	20.59
With diagnosis/no treatment	6555	62.1
With diagnosis/with treatment	1673	15.85
Missing data	154	1.46
Most important basis for tumor diagnosis		
Histology of the primary tumor	10 321	97.77
Other	175	1.66
Missing data	60	0.57
Histological type of primary tumor		
Adenocarcinoma	10 400	98.52
Other	156	1.48
Occurrence of more than one primary tumor		
No	10 049	95.2
Yes	320	3.03
Uncertain	28	0.27
Missing data	159	1.51
Clinical tumor staging by group (TNM)		
Stage I	735	6.96
Stage II	2545	24.11
Stage III	1017	9.63
Stage IV	1095	10.37
Missing data	5164	48.92
Distant metastasis		
No	9675	91.65
Yes	881	8.35
Surgery		
No	8053	76.29
Yes	2503	23.71
Radiotherapy		
No	8501	80.53
Yes	2055	19.47
Hormone therapy		
No	8237	78.03
Yes	2319	21.97
Radiotherapy combined with hormone therapy		
No	8957	84.85
Yes	1599	15.15
Disease status at the end of the first hospital treatment		
No evidence of disease (complete remission)	1880	17.81
Partial remission	243	2.3
Stable disease	1576	14.93
Disease progression	355	3.36
Oncologic supportive care	258	2.44
Death	785	7.44
Missing data	5459	51.71
Outcomes		
Survived at the end of the follow‐up period	6388	60.52
Died of PCa	1936	18.34
Died of other causes	2232	21.14

At the end of follow‐up, 18.34% of patients had died from PCa and 21.14% had died from other causes, indicating a relevant competing‐event structure that should be considered when interpreting Kaplan–Meier survival estimates.

A substantial proportion of missing data was observed for TNM staging (48.92%) and disease status at the end of treatment (51.71%), which limited their inclusion in multivariable modeling and required careful interpretation of adjusted estimates. Table 2 shows the PCa survival estimates using the Kaplan–Meier method and their respective 95% confidence intervals, as well as the *p*‐value of the log rank test for stratification. The PCa‐specific survival was 87.7% at 5 years, and significant differences (*p* < 0.05) were found for age group, race/color, marital status, educational level, referral source, prior diagnosis and treatment, clinical tumor staging by group (TNM), presence of distant metastases, and initial treatment received, including surgery, radiotherapy, and hormone therapy. For this univariate analysis, missing values for each specific variable were not considered.

**TABLE 2:** Prostate cancer‐specific survival (OS) and 95% confidence interval (95% CI) estimated by the Kaplan–Meier method for prostate cancer mortality at 5 years.

Variable	*N*	5‐year OS^a^		Log‐rank test
		OS	IC 95%	*p*‐
All population	10 556	0.877	0.871–0.884	—
Age range at diagnosis	10 556			< 0.001
18–64 years	3126	0.923	0.913–0.932	
65 years or older	7430	0.857	0.849–0.865	
Race/skin color	9704			0.040
White	3010	0.884	0.872–0.896	
No White	6694	0.872	0.864–0.881	
Marital status	10 024			0.010
Married	6959	0.884	0.876–0.892	
No married	3065	0.863	0.851–0.876	
Education level	7956			< 0.001
No education	1213	0.834	0.813–0.856	
Basic education	5505	0.879	0.870–0.888	
Middle or high education	1238	0.907	0.890–0.923	
Referral source	8882			0.040
SUS	7047	0.887	0.879–0.894	
No SUS	1835	0.863	0.847–0.879	
Previous diagnosis and treatment	10 402			< 0.001
No diagnosis/no treatment	2174	0.843	0.827–0.859	
With diagnosis/no treatment	6555	0.883	0.875–0.891	
With diagnosis/with treatment	1673	0.898	0.883–0.913	
Clinical tumor staging by group (TNM)	5392			< 0.001
Stage I	735	0.965	0.951–0.978	
Stage II	2545	0.93	0.920–0.941	
Stage III	1017	0.917	0.900–0.935	
Stage IV	1095	0.499	0.469–0.531	
Distant metastasis	10 556			< 0.001
No	9675	0.917	0.911–0.923	
Yes	881	0.425	0.392–0.460	
Surgery	10 556			< 0.001
No	8053	0.853	0.845–0.861	
Yes	2503	0.951	0.943–0.960	
Radiotherapy	10 556			< 0.001
No	8501	0.858	0.850–0.866	
Yes	2055	0.954	0.945–0.964	
Hormone therapy	10 556			< 0.001
No	8237	0.905	0.899–0.911	
Yes	2319	0.774	0.756–0.792	
Radiotherapy combined with hormone therapy	10 556			0.400
No	8957	0.874	0.867–0.881	
Yes	1599	0.893	0.877–0.908	

^a^ Prostate cancer‐specific survival (OS) and 95% confidence interval ([95% CI]) estimated by the Kaplan–Meier method for prostate cancer mortality.

**FIGURE 1:**
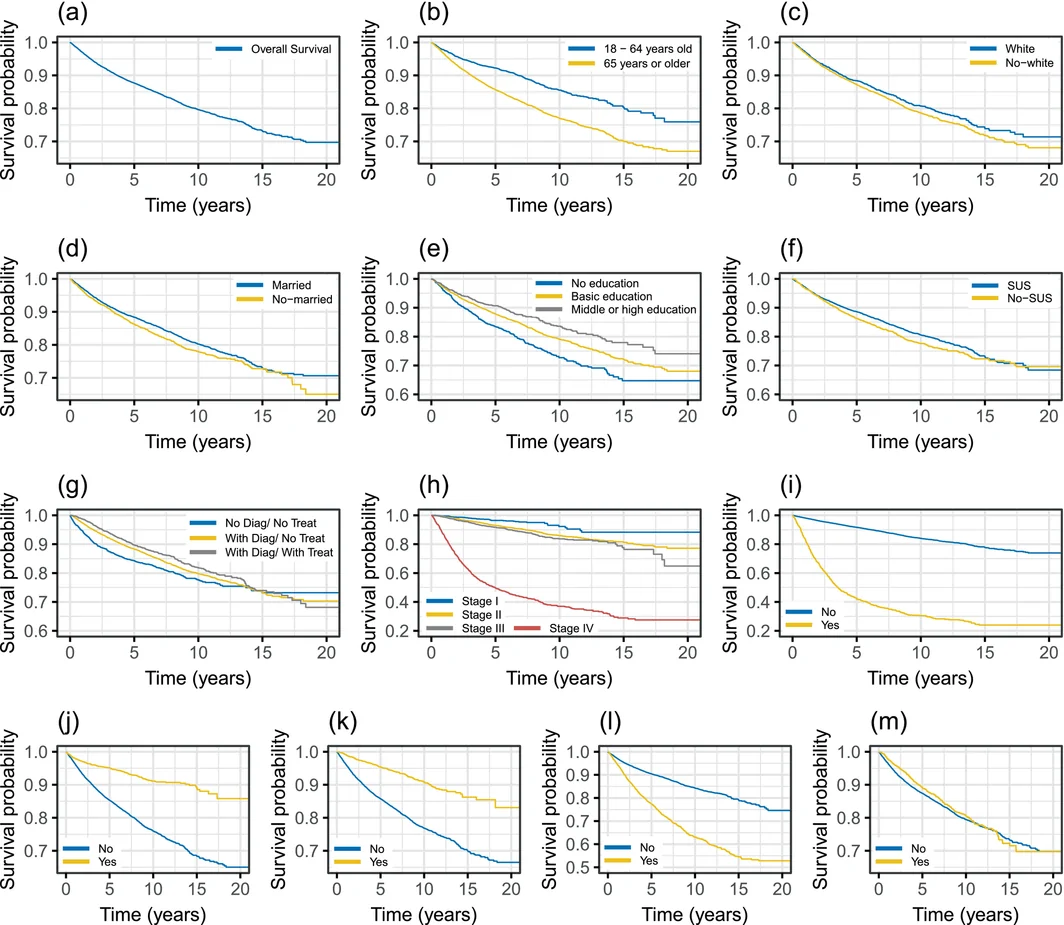
Kaplan–Meier prostate cancer survival curves for (a) all population, (b) age range at diagnosis, (c) race/skin color, (d) marital status, (e) education level, (f) referral source, (g) previous diagnosis and treatment, (h) clinical tumor staging by group (TNM), (i) distant metastasis, (j) first treatment: surgery, (k) first treatment: radiotherapy, (l) first treatment: hormone therapy, and (m) first treatment: radiotherapy and hormone therapy combined. Age was categorized for visualization purposes in Kaplan–Meier curves, whereas it was modeled as a continuous variable in the multivariable analysis. Kaplan–Meier curves were used to estimate overall survival patterns. However, because deaths from causes other than prostate cancer were treated as censored observations, these curves may overestimate the cumulative incidence of prostate cancer‐specific mortality in the presence of competing risks.

Figure 1 shows the Kaplan–Meier survival curves estimated for the entire population and stratified between the selected variables.

The proportional hazards assumption was first evaluated for all covariates included in the univariable analysis using Schoenfeld residuals. Some variables, including previous diagnosis and treatment, clinical tumor staging (TNM), distant metastasis, radiotherapy, and combined radiotherapy and hormone therapy, showed evidence of nonproportional hazards (*p* < 0.05), suggesting potential time‐dependent effects.

In the multivariable model, however, the Schoenfeld residual tests did not indicate significant violations of the proportional hazards assumption for any of the included covariates (Table 3), supporting the adequacy of the final model specification.

**TABLE 3:** Schoenfeld's test for all covariates in the model.

Variables	*χ* ^2^	df	*p*
Age at diagnosis	1.720	1	0.190
Race/skin color	0.127	1	0.720
Marital status	1.420	1	0.230
Education level	1.280	2	0.530
Origin of referral	1.550	1	0.210
Previous diagnosis and treatment	62.600	2	< 0.001
Clinical tumor staging by group (TNM)	65.600	3	< 0.001
Distant metastasis	65.000	1	< 0.001
Surgery	2.920	1	0.088
Radiotherapy	12.000	1	< 0.001
Hormone therapy	0.811	1	0.370
Radiotherapy combined with hormone therapy	13.900	1	< 0.001

**FIGURE 2:**
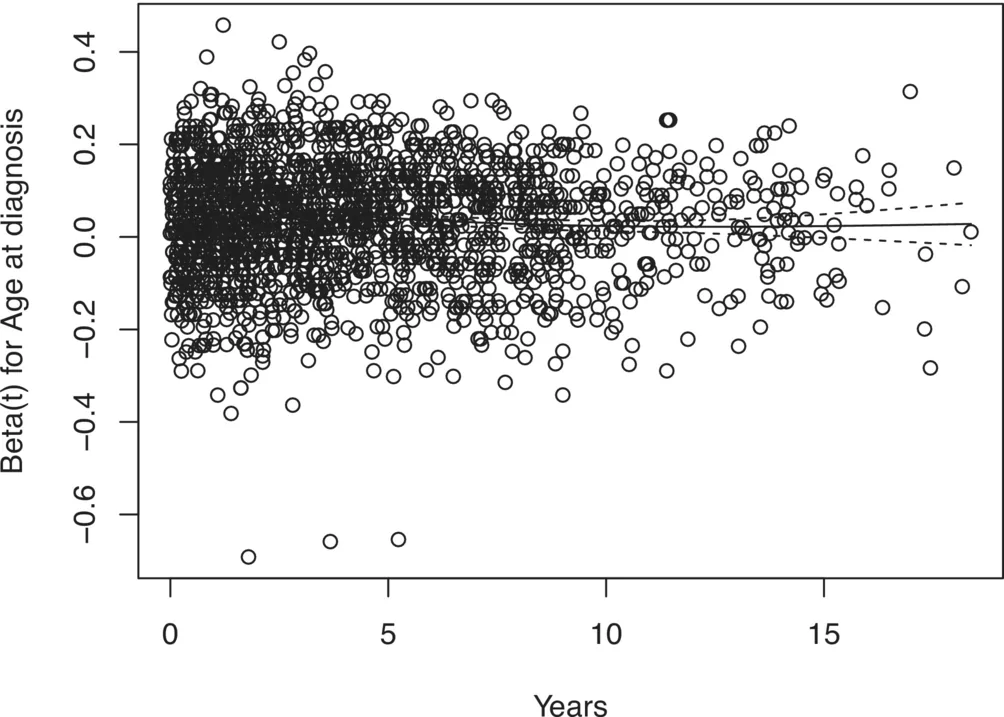
Schoenfeld's test for continuous variable age at diagnosis.

The continuous variable, age at diagnosis, showed proportionality of risks over time, as confirmed by the Shoenfeld residual test (*p* = 0.270), and as shown in Figure 2.

The sensitivity analysis comparing patients included in the multivariable Cox models (complete‐case sample; *n* = 6481) with those excluded due to missing data (4075) is presented in Table 4. Although several statistically significant differences were observed, likely due to the large sample size, most variables showed negligible or small imbalance according to SMDs. Key prognostic variables, including age at diagnosis (SMD = 0.009), race/skin color (SMD = 0.042), marital status (SMD = 0.016), distant metastasis (SMD = 0.042), and the study outcome (SMD = 0.086) were broadly comparable groups. More pronounced imbalances were observed mainly for variables directly affected by missing data patterns, especially previous diagnosis/treatment and occurrence of more than one primary tumor. Overall, these findings suggest that the complete‐case analysis was unlikely to have introduced substantial prognostic selection bias into the primary multivariable analysis.

**TABLE 4:** Comparison of baseline characteristics between patients included in the complete‐case multivariable Cox model and patients excluded due to missing data.

Variable	Complete‐case sample (*n* = 6481)		Excluded‐case sample (*n* = 4075)		*p*	Standardized mean difference test	
	*N*	%	*N*	%		SMD	Interpretation
Age at diagnosis (in years)					0.670^a^	0.009	Negligible imbalance
Mean (standard deviation)	69.17 (9.03)	—	63.25 (9.23)	—			
Median (interquartile range)	70 (63–75)	—	70 (63–76)	—			
Age at diagnosis					0.751^b^	0.007	Negligible imbalance
18–64 years	1927	29.73	1199	29.42			
65 years or older	4554	70.27	2876	70.58			
Race/skin color					0.052^b^	0.042	Negligible imbalance
White	1968	30.37	1042	32.33			
No White	4513	69.63	2181	67.67			
Marital status					0.463^b^	0.016	Negligible imbalance
Married	1965	30.32	1100	26.99			
No married	4516	69.68	2443	59.95			
Education level					0.001^b^	0.102	Small imbalance
No education	994	15.34	219	14.85			
Basic education	4524	69.80	981	66.51			
Middle or high education	963	14.86	275	18.64			
State of residence					< 0.001^b^	0.069	Negligible imbalance
Espírito Santo	6339	97.81	3940	96.69			
Other state	142	2.19	135	3.31			
Referral source					< 0.001^b^	0.190	Small imbalance
SUS	5012	77.33	2035	84.76			
No SUS	1469	22.67	366	15.24			
Previous diagnosis and treatment					< 0.001^b^	0.388	Meaningful imbalance
No diagnosis/no treatment	974	15.03	1200	29.45			
With diagnosis/no treatment	4248	65.55	2307	56.61			
With diagnosis/with treatment	1194	18.42	479	11.75			
Missing data	65	1.00	89	2.18			
Most important basis for tumor diagnosis					< 0.001^b^	0.177	Small imbalance
Histology of the primary tumor	6406	98.84	3915	96.07			
Other	57	0.88	42	1.03			
Missing data	18	0.28	118	2.90			
Histological type of primary tumor					< 0.001^b^	0.125	Small imbalance
Adenocarcinoma	6425	99.14	3975	97.55			
Other	56	0.86	100	2.45			
Occurrence of more than 1 primary tumor					< 0.001^c^	0.285	Meaningful imbalance
Yes	6265	96.67	3784	92.86			
No	200	3.09	120	2.94			
Uncertain	16	0.25	12	0.29			
Missing data	0	0.00	159	3.90			
Clinical tumor staging by group (TNM)					< 0.001^b^	0.154	Small imbalance
Stage I	461	7.11	274	6.72			
Stage II	1687	26.03	858	21.06			
Stage III	545	8.41	472	11.58			
Stage IV	695	10.72	400	9.82			
Missing data	3093	47.72	2071	50.82			
Distant metastasis					0.039^b^	0.042	Negligible imbalance
No	5911	91.21	3764	92.37			
Yes	570	8.79	311	7.63			
Surgery					< 0.001^b^	0.101	Small imbalance
No	5052	77.95	3001	73.64			
Yes	1429	22.05	1074	26.36			
Radiotherapy					< 0.001^b^	0.115	Small imbalance
No	5107	78.80	3394	83.29			
Yes	1374	21.20	681	16.71			
Hormone therapy					< 0.001^b^	0.072	Negligible imbalance
No	4983	76.89	3254	79.85			
Yes	1498	23.11	821	20.15			
Radiotherapy combined with hormone therapy					< 0.001^b^	0.101	Small imbalance
No	5410	83.47	3547	87.04			
Yes	1071	16.53	528	12.96			
Disease status at the end of the first hospital treatment					< 0.001^b^	0.189	Small imbalance
No evidence of disease	1265	19.52	615	15.09			
Partial remission	156	2.41	87	2.13			
Stable disease	958	14.78	618	15.17			
Disease progression	201	3.10	154	3.78			
Oncologic supportive care	114	1.76	144	3.53			
Death	411	6.34	374	9.18			
Missing data	3376	52.09	2083	51.12			
Outcomes					< 0.001^b^	0.086	Negligible imbalance
Survived at the end of the follow‐up period	3989	61.55	2399	58.87			
Died of prostate cancer	1210	18.67	726	17.82			
Died of other causes	1282	19.78	950	23.31			

*Note:* Values < 0.10 indicate negligible differences, 0.10–0.20 small differences, and > 0.20 meaningful imbalance between groups.

Abbreviation: SMD, standardized mean difference.

^a^ Two sample *t*‐test.

^b^ Pearson's *χ* ^2^ test.

^c^ Fisher's exact test.

In the multivariable Cox proportional hazards model, increasing age at diagnosis (per 10‐year increment) was independently associated with a higher risk of PCa‐specific mortality (HR = 1.14; 95% CI: 1.06–1.22; *p* < 0.001) (Table 5). Race/skin color and marital status were not significantly associated with mortality after adjustment.

**TABLE 5:** Multivariable analysis of the risk factors of prostate cancer mortality in patients with prostate cancer (*n* = 6481).

Variables		HR	95% CI	*p*
Age at diagnosis				
Every 10‐year increment		1.14	1.06–1.22	< 0.001
Race/skin color				
White		1	—	—
No White		1.03	0.96–1.17	0.572
Marital status				
Married		1	—	—
No married		1.09	0.91–1.23	0.150
Education level				
No education		1	—	—
Basic education		0.82	0.71–0.95	0.009
Middle or high education		0.64	0.52–0.80	< 0.001
Referral source				
SUS		1	—	—
No SUS		1.14	1.00–1.30	0.048
Surgery				
No		1	—	—
Yes		0.41	0.33–0.51	< 0.001
Hormone therapy				
No		1	—	—
Yes		1.98	1.75–2.23	< 0.001

Abbreviations: CI: confidence interval; HR: Hazard ratio.

Higher educational attainment was associated with a reduced risk of death, with basic education (HR = 0.82; 95% CI: 0.71–0.95; *p* = 0.009) and middle or high education (HR = 0.64; 95% CI: 0.52–0.80; *p* < 0.001) showing protective effects compared to no formal education.

Referral source was also associated with mortality, as patients referred from non‐SUS services had a modestly higher risk of PCa‐specific death compared to those from the public system (HR = 1.14; 95% CI: 1.00–1.30; *p* = 0.048).

With regards to treatment variables, surgery was associated with a substantially lower risk of mortality (HR = 0.41; 95% CI: 0.33–0.51; *p* < 0.001), whereas hormone therapy was associated with an increased risk of PCa‐specific death (HR = 1.98; 95% CI: 1.75–2.23; *p* < 0.001) (Table 5).

## 4 Discussion

This study aimed to estimate survival and investigate factors associated with mortality among men diagnosed with PCa who received care within the Cancer Care Network of a Brazilian state between 2000 and 2016. We found that the PCa‐specific survival is 87.7% in 5 years.

Comparatively, using data from the Brazilian Unified Health System (SUS), a national cohort showed a survival rate of 83% at 5 years [35], while a study held in Mato Grosso, Brazil, found a survival rate of 79.6% at 5 years [20]. Another earlier national study, with data from the National Oncology Base from 2001 to 2009, showed a PCa survival rate of only 70% at 5 years [36], lower than other findings. In developed countries, advances in cancer diagnosis and treatment, particularly PCa, may result in a greater proportion of cured patients or increased survival, as seen in studies in the United States, where PCa survival at 5 years was 96.7% [37] and 98% [6, 8].

An important risk factor at the time of diagnosis of prostate neoplasia is increasing age [16]. Here, we found that 5‐year survival was statistically lower in patients aged 65 years or older compared to those < 65 years. Furthermore, multivariable analysis showed that each additional 10 years at diagnosis increased the risk of death from PCa by 14%. This finding is consistent with other studies of men with PCa in other countries [38, 39].

The variables race/skin color and marital status present survival with statistically significant differences, indicating lower PCa survival rates for non‐white and non‐married patients compared to white and married patients; however, this difference was not reflected in a greater risk of death from PCa when adjusted by multivariable Cox regression. On the other hand, these variables are potential predictors of PCa mortality risk [40, 41, 42, 43].

Our univariate analysis showed a reduction in survival for lower levels of education. In the multivariable model, education level remained an independent predictor of PCa‐specific mortality, with a clear gradient showing lower risk among individuals with higher educational attainment. Low education is associated with late diagnosis and treatment, which reduces patients' chances of cure and increases their mortality [29, 44]. In addition, patients' delay in seeking health care may be due to lack of information and access and/or lack of understanding of their current health status [45].

Late diagnosis is generally associated with advanced tumor stage, which indicates a less favorable prognosis and higher PCa mortality from PCa [8, 10]. In our study, PCa‐specific survival decreased with more advanced clinical tumor stage at diagnosis, which is consistent with studies in national cohorts [35, 36] and worldwide [39, 46, 47]. The presence of metastases was associated with a more than 50% reduction in PCa survival at 5 years compared to patients with non‐metastatic cancer [47]. In addition to a worse prognosis, metastatic PCa is associated with significant morbidity, need for supportive care, and poor quality of life for patients [19, 47, 48].

In our study, patients who received surgery and radiotherapy as initial treatment had higher survival rates and lower specific risk of death compared to those who did not receive these treatments. The observed associations between treatment modalities and PCa‐specific mortality must be interpreted within the clinical context in which treatment decisions are made. In real‐world settings, patients selected for surgery or radiotherapy are typically those with localized disease, preserved functional status, and eligibility for curative‐intent strategies [48, 49, 50]. Consequently, the lower mortality observed in these groups is more likely to reflect favorable baseline prognostic characteristics rather than a direct protective effect of the interventions themselves.

In contrast, patients managed without curative treatments—or those receiving systemic therapies—often present with more advanced disease or clinical conditions that limit therapeutic options, as reflected in current guideline recommendations for PCa management [48, 49, 50, 51]. These differences in baseline clinical characteristics introduce an important source of confounding in observational studies, particularly, confounding by indication, since treatment allocation is strongly influenced by disease severity and patient eligibility for curative‐intent therapies [15].

In our study, this issue is further accentuated by the absence of complete TNM staging information, which restricted our ability to fully account for disease severity in the adjusted models. Therefore, the associations between treatment modalities and mortality outcomes should be interpreted as reflecting underlying clinical selection processes rather than causal treatment effects. This interpretation is consistent with prior analyses conducted in the same population using competing‐risk methods, which demonstrated that treatment‐related associations are strongly influenced by baseline disease severity and clinical selection patterns [52].

When estimating PCa mortality using the cause‐specific Cox model, it is important to acknowledge that deaths from other causes act as competing risks. It is important to emphasize that cause‐specific Cox and Fine–Gray models address different, although complementary, research questions [22]. While Fine–Gray models are more appropriate for estimating cumulative incidence in the presence of competing risks, cause‐specific Cox models are particularly useful for etiological analyses because they estimate the effect of covariates on the instantaneous hazard of the event of interest [31]. Therefore, the present findings should be interpreted within an etiological framework rather than as direct estimates of absolute cumulative risk. We also acknowledge that, because deaths from other causes were frequent in this cohort, Kaplan–Meier curves may overestimate the cumulative probability of PCa‐specific death [30]. For that reason, these curves were retained as descriptive survival displays, whereas the multivariable interpretation was based on the cause‐specific hazard model.

The present findings should be interpreted as complementary to, rather than duplicative of, our previous competing‐risk analysis conducted using the same population‐based cohort [22]. Although both investigations evaluated PCa mortality, they were designed to answer fundamentally different epidemiological questions through distinct statistical frameworks. The previous study focused on estimating the cumulative incidence of PCa‐specific mortality and subdistribution hazard ratios using the Fine–Gray competing‐risk model [22], whereas the present investigation was specifically designed to evaluate prognostic factors associated with the cause‐specific hazard of PCa mortality using cause‐specific Cox regression [31]. Accordingly, the use of the same cohort should be regarded as a methodological strength, as it enables direct comparison between complementary analytical frameworks while avoiding population heterogeneity that could otherwise influence the interpretation of prognostic associations.

These methodological differences have important implications for the interpretation of the results. Cause‐specific hazard ratios quantify the instantaneous rate of PCa death among individuals who remain event‐free during follow‐up and are particularly informative for etiological inference and identification of prognostic determinants [31]. In contrast, Fine–Gray subdistribution hazard ratios estimate how prognostic factors influence the cumulative probability of PCa‐specific death in the presence of competing mortality [22]. Consequently, although similar variables may appear associated with mortality in both studies, the resulting estimates should not be interpreted interchangeably because they represent different dimensions of disease prognosis.

Taken together, these complementary analytical approaches provide a broader understanding of long‐term PCa outcomes than either method alone. Competing‐risk models are particularly valuable for estimating absolute prognosis and supporting population‐level risk prediction, whereas cause‐specific Cox models are better suited for investigating etiological relationships and prognostic mechanisms. Accordingly, the present study provides complementary scientific evidence that could not be obtained from the competing‐risk framework alone and expands the epidemiological interpretation of PCa survival within the Brazilian oncology care network.

A major limitation of this study is the substantial proportion of missing data in key clinical variables, particularly TNM staging and disease status after treatment. The absence of TNM staging in the multivariable model may have introduced residual confounding, especially in the interpretation of treatment‐related variables. For instance, the association observed between hormone therapy and increased PCa mortality likely reflects confounding by indication, as this treatment is typically administered to patients with more advanced disease stages, which could not be fully accounted for due to missing staging data.

Although multiple imputation could be considered, the high proportion of missingness and the likely non‐random nature of missingness would require strong and potentially unverifiable assumptions. Therefore, we opted for a complete‐case analysis, acknowledging the potential for selection bias and reduced statistical power. Additionally, the Kaplan–Meier curves presented in this study should be interpreted with caution in the context of competing risks. Because deaths from causes other than PCa were treated as censored observations, Kaplan–Meier estimates may overestimate the cumulative incidence of PCa‐specific mortality. Accordingly, these curves should be interpreted as descriptive survival estimates rather than direct cumulative incidence estimates.

Another important limitation is the absence of key clinical prognostic variables, including Gleason score, prostate‐specific antigen (PSA) levels, risk stratification categories, and treatment intent (curative vs. palliative). These factors are central to PCa prognosis and clinical decision‐making, and their unavailability in the dataset limits the ability to fully adjust for disease severity. Consequently, residual confounding cannot be excluded, particularly in the interpretation of treatment‐related associations. Given that treatment selection is strongly influenced by these clinical parameters, the observed associations between therapeutic modalities and mortality should be interpreted as reflecting underlying disease severity and clinical decision pathways rather than causal treatment effects. Similar limitations have been reported in previous population‐based studies of PCa outcomes [50, 51, 52, 53].

## 5 Conclusions

The present study evaluated survival and factors associated with PCa‐specific mortality among patients diagnosed between 2000 and 2016 and treated within the oncology care network of the state of Espírito Santo, Brazil. The 5‐year PCa‐specific survival was 87.7%. Increasing age, lower educational level, presence of distant metastasis at diagnosis, and referral from non‐SUS services were independently associated with a higher risk of mortality.

Regarding treatment‐related variables, surgery was associated with a lower risk of death, whereas hormone therapy was associated with higher mortality. However, these associations should be interpreted with caution, as they likely reflect underlying disease severity and treatment selection patterns rather than causal treatment effects.

These findings underscore the importance of early diagnosis, reduction of social inequalities in access to care, and strengthening of integrated oncology services. From a public health perspective, improving the quality and completeness of clinical data in cancer registries is also essential to enhance the validity of survival analyses and support evidence‐based decision‐making.

Finally, the complementary application of cause‐specific survival analysis and competing‐risk methods illustrates how distinct statistical frameworks can answer different but complementary epidemiological questions, providing a more comprehensive understanding of PCa prognosis for future epidemiological research and clinical decision‐making.

## Author Contributions

**Luciane Bresciani Salaroli:** writing – original draft, writing – review and editing, validation, data curation. **Wesley Rocha Grippa:** conceptualization, validation, software, data curation, formal analysis, writing – original draft, writing – review and editing. **Cristiano Soares Dell'Antônio:** writing – original draft, writing – review and editing, visualization, data curation. **Larissa Soares Dell'Antonio:** validation, visualization, writing – review and editing, writing – original draft, data curation. **Luís Carlos Lopes‐Júnior:** conceptualization, methodology, software, data curation, investigation, validation, formal analysis, supervision, funding acquisition, visualization, project administration, resources, writing – original draft, writing – review and editing.

## Funding

Fundação de Amparo à Pesquisa e Inovação do Espírito Santo (FAPES), under the public call FAPES No. 13/2025—UNIVERSAL, Grant Agreement No. 1599/2025, Process number: 2025‐4NJHN.

## Conflicts of Interest

The authors declare no conflicts of interest.

## Data Availability

The data that support the findings of this study are available on request from the corresponding author. The data are not publicly available due to privacy or ethical restrictions.
